# Construction of Gene Regulatory Networks Using Recurrent Neural Networks and Swarm Intelligence

**DOI:** 10.1155/2016/1060843

**Published:** 2016-05-19

**Authors:** Abhinandan Khan, Sudip Mandal, Rajat Kumar Pal, Goutam Saha

**Affiliations:** ^1^Department of Computer Science and Engineering, University of Calcutta, Acharya Prafulla Chandra Roy Siksha Prangan, JD-2, Sector III, Salt Lake City, Kolkata, West Bengal 700 098, India; ^2^Department of Information Technology, North Eastern Hill University, Umshing-Mawkynroh, Shillong, Meghalaya 793 022, India

## Abstract

We have proposed a methodology for the reverse engineering of biologically plausible gene regulatory networks from temporal genetic expression data. We have used established information and the fundamental mathematical theory for this purpose. We have employed the Recurrent Neural Network formalism to extract the underlying dynamics present in the time series expression data accurately. We have introduced a new hybrid swarm intelligence framework for the accurate training of the model parameters. The proposed methodology has been first applied to a small artificial network, and the results obtained suggest that it can produce the best results available in the contemporary literature, to the best of our knowledge. Subsequently, we have implemented our proposed framework on experimental (*in vivo*) datasets. Finally, we have investigated two medium sized genetic networks (*in silico*) extracted from GeneNetWeaver, to understand how the proposed algorithm scales up with network size. Additionally, we have implemented our proposed algorithm with half the number of time points. The results indicate that a reduction of 50% in the number of time points does not have an effect on the accuracy of the proposed methodology significantly, with a maximum of just over 15% deterioration in the worst case.

## 1. Introduction

With the ongoing evolution of technology, massive amounts of temporal genetic expression data for different diseases are becoming available to researchers. The analysis of these data can potentially reveal many unknown cellular activities of living organisms [1, 2]. These data have enough hidden information embedded in them that if suitably analysed can revolutionise biological science and its allied applications like drug design. Accordingly, this has attracted and motivated the research fraternity to undertake detailed investigations in this domain and subsequently develop computational tools required for biologically credible analysis of these data [3–6]. In this paper, we have examined the reverse engineering of gene regulatory networks (GRNs) from temporal genetic expression datasets. These types of datasets contain crucial underlying information concerning the network dynamics among the genes (through protein).

**Figure 1:**
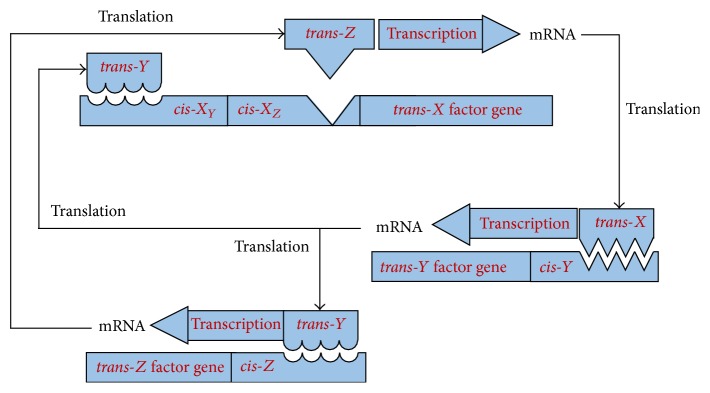
Gene regulation with both positive and negative feedback.

A GRN represents the complex interregulatory relationships among genes. The transcriptional regulation of genes by other genes involves DNA, RNA, and protein as well as other molecules. The genetic interactions are indirect; that is, a gene does not interact with other genes directly. The indirect interactions take place with the help of proteins (a.k.a. transcription factors). The regulatory relationships (depending on the nature of the control) may be of two types, namely,* activation* (where there is an increase in the expression value of the target gene) and* repression* (where the expression value of the target gene decreases). The various processes involved in genetic regulation have been shown in Figure 1.

Genetic expression datasets deal with the expression values of a vast number of interacting genes. Moreover, the number of genes in a dataset is generally two to three times more than the number of time points, at the very least. This imposes a well-known computational problem known as the* curse of dimensionality* [7]. Another difficulty imposed by microarray datasets is considerable noise contamination [8]. The current work deals with small to medium sized networks and hence is not faced with the former problem. However, the authors do focus on the performance of the proposed methodology in the presence of noise.

In this paper, we have proposed a new methodology for the accurate extraction of the topology of a GRN from any given noisy temporal genetic expression dataset using a statistical paradigm based on the theory of combination. The methodology has an underlying hybrid swarm intelligence framework which is basically a* Bat Algorithm* (BA) inspired* Particle Swarm Optimization* (PSO) algorithm christened BAPSO by the authors. Here, better results have been obtained compared to the contemporary literature for the benchmark networks considered. The proposed methodology uses the* Recurrent Neural Network* (RNN) for modelling the required network dynamics.

According to Bolouri and Davidson [9], a gene in a GRN is usually regulated by 4 to 8 other genes. We have proposed a novel GRN construction strategy (based on this concept) that generates candidate architectures with a limit to the maximum number of regulators for each gene in the network. Since, in this work, we have studied only small-scale and medium-scale networks, we have assumed the maximum number of regulators to be 4 [9]. The fundamental mathematical theory of combination has been applied to search all the candidate solutions in the discrete search space of network constructions exhaustively. The corresponding RNN model parameters have been trained by the proposed hybrid metaheuristic technique that can replicate the original network dynamics faithfully. The quality of a solution architecture depends on the quantum of error in the predicted dynamics. The authors have observed in this investigation that biologically plausible candidate architectures return much-reduced prediction errors compared with those which are far removed from real-world network structures.

We have implemented our proposed algorithm on three different types of data:

- A synthetic dataset generated from an artificial network which has been studied quite extensively concerning reverse engineering of GRNs.
- A real-world experimental dataset (*in vivo*), that is, the DNA SOS repair network of* E. coli*.
- An artificial dataset generated* in silico* from a real-world network of* E. coli*.
- Another artificial dataset generated* in silico* from a real-world network of* yeast*.

Also, we have incorporated networks from small to medium scale in this work (i.e., 4-gene to 20-gene networks). In the case of the synthetic dataset, GRNs predicted by our proposed methodology generate improved results concerning the prediction of correct as well as incorrect regulations, compared to the best existing results in the contemporary literature (to the best of our knowledge). In the case of the* in silico* experiments, the results suggest that our proposed algorithm is robust enough to return fewer incorrect predictions along with an increase in the number of correct predictions compared to the best available outcomes in recent research endeavours. For the real experimental datasets, it has been observed that the proposed methodology can identify all the possible gene regulatory relations, some of which are quite elusive to the contemporary as well as previous researchers.

The rest of the paper has been organized as follows. The background of temporal genetic expression dataset study has been presented in the next section with an outline of the existing methodologies for reverse engineering of GRNs. The subsequent section presents our proposed framework in detail. Experimental results have been presented and discussed next. The final section concludes the paper, highlighting some future research scopes.

## 2. Preliminaries

### 2.1. Background

Traditional investigations in the domain of molecular biology provide vital information about the functioning of the genetic system in a living cell. Regrettably, this information so far is inadequate for us to comprehend the complex gene regulatory mechanisms fully. Among such techniques, southern blotting was first reported by Augenlicht and Kobrin [10], and it is the origin of DNA microarray technology [11]. Improvements in this technology have allowed us to measure the expression levels of thousands of genes simultaneously under various circumstances. These data help us in the pursuit of disclosing the knowledge about the regulatory interactions between genes of the entire genome of a living organism. Despite these advancements, there remain numerous open challenges in system biological research domain.

Various approaches exist in the contemporary literature for the construction of GRNs from time series genetic expression datasets. At the outset, researchers attempted to employ clustering algorithms on temporal expression data based on pairwise correlation coefficients [12] and Euclidian distances [13] for the reconstruction of GRNs. Information theory based approaches that made use of “mutual information” between different expression profiles have also been implemented by researchers for defining similarity between genes [14–16]. Application of Bayesian networks for modelling of GRNs is also quite popular among the researcher fraternity [17–20].

GRNs can be effectively constructed using the dynamical modelling formalisms [21] such as Boolean networks [22], where Boolean variables are used to represent the interaction between genes, and the ordinary differential equations based method, *S*-systems [23–25], where in-depth biochemical kinetic models are used to simulate gene network architectures. All of the above can reproduce the structure as well as the temporal expression profiles from temporal genetic expression profiles.

**Figure 2:**
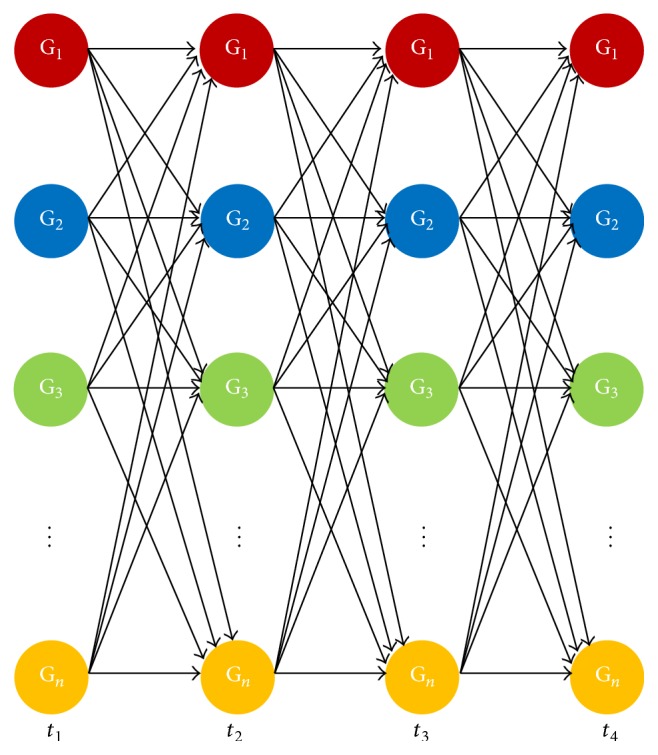
RNN model description of a genetic network. The network shown is unfolded from *t* = *t*_1_ to *t* = *t*_4_. Here, all possible connections have been shown among the genes whereas, in reality, such networks are only sparsely connected.

Additive regulation networks have also been used by researchers to represent the dynamics of a GRN [26]. The collective regulatory effect of a group of genes on a target gene can be represented in this formalism. The intensity and type of a particular interaction between a target (*i*) and a regulator (*j*) are defined by *w*_*ij*_: a positive value denotes expression (facilitation) and a negative value denotes repression while a zero (0) value implies that there is no interaction between *i* and *j*. Thus, a GRN can be represented by a weight matrix *W* = [*w*_*ij*_]_*N*×*N*_, where the number of genes in the GRN is equal to *N* [27, 28]. Another model somewhat analogous to this model is the* Recurrent Neural Network* (RNN) model, which has been effectively used in the reconstruction of GRNs from temporal expression data by several contemporary researchers [29–35]. The theoretical background of the RNN formalism has been discussed in detail in the next section. This forms the basis of our proposed modified framework.  Figure 2 shows the representation of a GRN by an RNN model.

### 2.2. Recurrent Neural Networks

The regulation of the expression of any particular gene, by another gene or a group of genes, can be expressed with the help of the Recurrent Neural Network formalism [30, 36–38] as shown in Figure 2. Each node symbolises a particular gene and the edges between the nodes represent the regulatory interactions among the genes. Each tier of the neural network defines the genetic expression level of the genes at a specified time *t*_*i*_. The level of expression of any particular gene at a time *t*_*i*+1_ = *t*_*i*_ + *dt* depends upon the genetic expression level of all the genes (*x*_*j*_) at the preceding time *t*_*i*_ and the weights of their corresponding connecting edges (*w*_*i*,*j*_) with that particular gene. Thus, the total regulatory effect of all the genes in a network, on any gene *i*, can be summarised as follows:

(1) $$\begin{array}{c} g_{i}=\overset{n}{\underset{j=1}{\sum }}w_{i,j}x_{j}+\beta_{i}. \end{array}$$

This can be transformed using a sigmoid function, within an interval [0,1], as has been shown by Vohradsky [31]. Here, *β*_*i*_ symbolises an external input, which may be visualised as a reaction delay parameter. A higher (large) value of this parameter indicates a reduction of the effect (influence) of *w*_*i*,*j*_ on *g*_*i*_. The actual genetic expression rate is subsequently modulated by a multiplicative constant *χ*_1_ that defines the peak expression level of a particular gene [31]. The rate of expression of any gene *i* can be defined as the total of the regulatory effects of other genes on it *δ*_*i*_ minus its degradation *γ*_*i*_. This is represented by

(2) $$\begin{array}{c} \frac{{dy}_{i}}{dt}=\delta_{i}-\gamma_{i}, \end{array}$$

where the degradation factor *γ*_*i*_ can be modelled based on the kinetic framework of a first-order biochemical equation represented as *γ*_*i*_ = *χ*_2*i*_ · *y*_*i*_ [31]. The term *δ*_*i*_ represents the entire regulatory effect on the expression of the gene, *i* (represented as *δ*_*i*_ = *χ*_1*i*_ · *f*(*g*_*i*_)). The constant *χ*_2_ signifies the rate constant of degradation of the gene product *i*. Thus,

(3) $$\begin{array}{c} \frac{{dy}_{i}}{dt}=\chi_{1i}\cdot f\left(\;\overset{n}{\underset{j=1}{\sum }}w_{i,j}x_{j}+\beta_{i}\;\right)-\chi_{2i}y_{i}, \end{array}$$

where *f* denotes the sigmoid transfer function and *x*_*j*_ signifies the concentrations of the elements of the given system (for *j* = *i*; *x*_*j*_ = *y*_*i*_). The above expresses the dynamics of expression of a gene *i* and denotes a “node” function [31]. Each node can be connected with all the other nodes to form a neural network (Figure 2). The weight matrix *w* describes the connection between the nodes of the network; a nonzero value of *w*_*ij*_ means that a connection between nodes *i* and *j* exists. The magnitude of the weight *w*_*ij*_ signifies the strength of an interaction, or a regulatory effect, between the two nodes. The neural network is completely defined by the differential equations respective to the particular nodes, and the number of equations is determined by the number of nodes. The quantum of genetic expression at any arbitrary time *t* can be calculated by solving the set of differential equations. Equation (3) represents a special case of a class of RNNs described by the more general equation:

(4) τidxidt=fi∑j=1nwi,jxj+βi−xi,

(5) τidxidt=11+exp⁡∑j=1nwi,jxj+βi−xi.

This is a continuous model that has been used for modelling brain activity pattern and the study of its dynamics. If the weight matrix *w*_*i*,*j*_ is symmetrical in nature, the network it represents reaches stability in finite time. Now, in real world, time series data are obtained at discrete time points only for which (5) can be rewritten in its discrete format as follows:

(6) τixit+Δt−xitΔt=11+exp⁡−∑j=1Nwi,jxj+βi−xit,or  xit+Δt=Δtτi1+exp⁡−∑j=1Nwi,jxj+βi−1−Δtτixi.

The dynamics of a GRN can be parametrically modelled using appropriate dynamical methodologies such as Bayesian networks, Boolean networks, Recurrent Neural Networks, and *S*-systems. This indicates the significance of identification of the underlying information regarding genetic interactions present in the temporal expression data of a regulatory network. The purpose of any reverse engineering framework is the accurate inference of the applied model's parameters for the faithful reproduction of the given time series data. This can be viewed as an optimization problem, where the model parameters are trained to minimise the difference between the simulated and the original time series data. Determination of the* mean square error* (MSE) from the above can be a suitable measure of this specification:

(7) $$\begin{array}{c} MSE=\frac{1}{NT}\overset{N}{\underset{i=1}{\sum }}\;\overset{T}{\underset{t=1}{\sum }}{\left(\;x_{i}\left(\;t\;\right)-{\tilde{x}}_{i}\left(\;t\;\right)\;\right)}^{2}. \end{array}$$

Here, *N* is the total number of genes (nodes) in the network, *T* is the total number of time points available, *x*_*i*_(*t*) is the original expression data, and ${\tilde{x}}_{i}(t)$ is the simulated data at any point of time *t*.

### 2.3. Major Concerns

One of the major hurdles in the reverse engineering of GRN from temporal gene expression data is the* curse of dimensionality*. It arises from the fact that the number of genes in a dataset is usually two to three orders higher than the number of time points, and it severely reduces the prediction capacity of the given formalisms. Researchers have attempted to solve this problem to some extent in [28, 30, 32, 33, 39, 40]. The present work focuses on small- to medium-scale networks only (4 genes to 20 genes) and thus does not face the entire severity of this problem.

The RNN methodology has been implemented, in this paper, to model the temporal expression data. For that purpose, the RNN model parameters require training, which, in essence, is an optimization problem. Several metaheuristic techniques, like Simulated Annealing [30, 41], Genetic Algorithm (GA) [32, 33, 40, 42, 43], Differential Evolution [44], Particle Swarm Optimization [34, 45, 46], and so forth, have been and are being implemented for this purpose with various levels of accuracy. The proposed methods, however, have largely been ineffective to accurately infer even small-scale real-life GRNs. A few have been able to identify all the true regulations but in the process have also inferred unwanted false regulations. Moreover, the “No Free Lunch” (NFL) theorem [47] rationally states that there is no single metaheuristic that is most appropriate for solving all types of optimization problems. Therefore, finding out the most suitable and efficient optimization techniques for the accurate inference of small GRNs is still an open problem for researchers.

Nevertheless, the number of parameters in need of training undergoes quadratic scaling with respect to the number of genes in a GRN. This fact imposes severe hindrance in keeping the dimension of the optimization problem at a reasonable computational limit. As a result, optimization of model parameters becomes implausible for practical values of *N* (i.e., *N* = 100, 1000, etc.). To solve this difficulty, researchers have proposed strategies like decomposition of the problem of global optimization of parameters into local problems of parameter optimization for a single target gene only [40, 43, 48, 49]. Other strategies, such as interpolation [36, 50, 51], the addition of noisy duplicate copies [52], and use of suitable thresholds [37, 52], have also been implemented to limit the number of optimizable parameters. Interpolation strategies usually have a drawback: they are incapable of faithfully summarising the dynamics between any two time points. According to van Someren et al., such strategies can bring about only a minimal reduction in the dimension of the optimization problem, irrespective of the number of additional time points [52]. Other strategies also fail to improve upon the situation as they also fail to add any supplementary information to the network dynamics.

Fortuitously, extensive biological research, in the perspective of reverse engineering of GRNs, confirms that there exist only a handful of genes that act as regulators in a GRN [7, 9, 19, 36, 37]; that is, GRNs are connected sparsely. Mathematically, this implies that we can assign a zero value to a large number of the model parameters that represent the one-on-one regulatory relationships. Thus, a considerable reduction in the dimensionality of the optimization problem can be achieved.

Researchers strived to develop a suitable optimization environment integrating this* sparseness concept* and achieve a significant improvement in the problem solution. This entailed some form of architectural constraint to be imposed on the predicted networks. Researchers have also found that it is possible to decouple the structural and the dynamic aspects of the given reverse engineering problem. In other words, there is scope for the application of a suitable technique that can decouple the problem into two independent subproblems: the search for candidate architectures in the discrete search space of network structures and the search for suitable model parameters in the corresponding continuous search space of parameters of dynamical formalisms [34, 35, 45, 46, 53, 54]. Thus, an endeavour to locate suitable model parameters may supervise the pursuit of detection of the candidate networks. The accuracy of a trained model, assessed from the perspective of reproducing the original dynamics, determines the appropriateness of a predicted architecture. The level of precision can be ascertained from the MSE calculated for the predicted models as per (7). A genetic interaction is appended to a predicted architecture or removed from it based on the value of the calculated MSE. Thus, the extra burden of searching the biologically plausible network architectures within a separate discrete search space of candidate network architectures is compensated by a considerable reduction in the dimension of the problem of training the dynamic model parameters.

## 3. Methods

### 3.1. Decoupled Strategy

In this section, we have explained in detail the methodology implemented in this work. Firstly, we have represented a GRN with the help of a directed graph; *G* = (*V*, *E*) represents a GRN, where *V* denotes the set of all nodes (*genes*) and *E* is the set of all edges (*the interaction between a pair of genes*). An edge, *e*_*i*,*j*_, is present in the set *E* if and only if there exists an interaction between node (*gene*) *i* and node (*gene*) *j*. Here, *e*_*i*,*j*_ signifies that gene *j* regulates gene *i*, and this convention has been used right through this work. A directed graph can be represented by an adjacency matrix for computational purposes. An adjacency matrix *G* = [*g*_*i*,*j*_]_*N*×*N*_, where *N* is the number of nodes in the graph (i.e.,* the number of genes in the network*). The element *g*_*i*,*j*_ can take any value, 0 or 1, depending on the absence or presence of a directed edge from node *j* to node *i*, respectively.

Now, the methodology proposed in this work, for the reverse engineering of GRNs from temporal expression datasets, employs the decoupling strategy discussed in the previous section [34, 35, 45, 46, 53, 54]. Here, we have first reduced the search space of candidate network structures by restricting the number of regulators [9] on a particular gene in a GRN. Subsequently, we have implemented the theory of combination to exhaustively search the reduced candidate network architecture space. In other words, if there are *N* genes in a GRN and *m* is the maximum number of regulators allowed for a gene, then the search space dimension is, by definition, _ _^*N*^ *C*_*m*_^ ^ or $\left(\begin{array}{c} N\\ m \end{array}\right)$. This is much less than the original search space dimension of 2^*N*^. Additionally, since our proposed algorithm is performing exhaustive search in the reduced space, it has a high chance of obtaining biologically plausible candidate network architectures. Mathematically, there are _ _^*N*^ *C*_*m*_^ ^ GRN structures, each represented by *G*_*i*_ (*i* = 1,2,…, _ _^*N*^ *C*_*m*_^ ^).

In the next phase, the RNN formalism has been implemented to model the underlying dynamics from the temporal genetic expression profiles based on the candidate network structures obtained in the previous phase. In other words, the weight matrix *W*_*i*_ of the RNN formalism has been initialised based on all *G*_*i*_'s defined. We have used the proposed BAPSO technique to train the RNN model parameters, that is, *w*_*ij*_, *β*_*i*_, and *τ*_*i*_, accurately such that the predicted expression profiles match the original expression profiles faithfully. The MSE defined by (7) determines the quality of a candidate solution *G*_*i*_, and the candidate solution with the least MSE has been chosen as the most reasonable from all the _ _^*N*^ *C*_*m*_^ ^ or $\left(\begin{array}{c} N\\ m \end{array}\right)$ candidates.

It would be interesting to note here that each of the genes in a GRN may not always be regulated by the maximum number of allowed regulators; that is, *m* = 4 genes. Therefore, we have gradually incremented the value of *m* from 1 to 4, and the MSE has been calculated for each case. A satisfactorily low value of MSE ~10^−3^ has been used as the selection criterion for a candidate solution.

A further problem encountered in this endeavour is the dimensionality of the RNN model parameter training problem. For *N* genes in a GRN, there are *N* × (*N* + 2) parameters to be trained for a particular RNN instance with the help of the BAPSO technique, and this essentially becomes computationally unrealistic for large values of *N*. To further reduce the computational load, in this work, we have decomposed this problem into *N* subproblems where, in each subproblem, (*N* + 2) parameters are trained for each of the *N* genes, independently. In case of each of the independent subproblems, the aim is to minimise the error term er_*i*_ defined as

(8) $$\begin{array}{c} {er}_{i}=\frac{1}{T}\overset{T}{\underset{t=1}{\sum }}{\left(\;x_{i}\left(\;t\;\right)-{\tilde{x}}_{i}\left(\;t\;\right)\;\right)}^{2}. \end{array}$$

Here, er_*i*_ ∈ *E* and *E* = [er_*i*_]_1×*N*_ which is subsequently used for the calculation of MSE. Hence,

(9) $$\begin{array}{c} MSE=\frac{1}{N}\overset{N}{\underset{i=1}{\sum }}{er}_{i}. \end{array}$$

The MSE governs the overall quality of the candidate solutions. The lower the value of the term er_*i*_, the more efficient the reduction in the difference between the predicted temporal expression profile and the original one and the more suitable the candidate network architecture. It is, therefore, the ultimate objective of the proposed methodology to reconstruct a network architecture that is biologically plausible and at the same time capable of replicating the original network dynamics more accurately.

### 3.2. The Proposed Metaheuristic

The training of the model parameters of the RNN instances has been achieved using the proposed BAPSO algorithm. Among all the proposed swarm intelligence techniques to date, Particle Swarm Optimization (PSO) [55–58] is conspicuous for being simple yet efficient, robust, easily tractable, and easy to code. PSO yields solutions that are of the same or better quality compared to GA for a wide array of problems and possesses a faster convergence rate. A particle swarm comprises some particles arbitrarily dispersed in a search space. The positions of these individual particles denote candidate solutions. The intention of any particle is to find the optimum solution utilising the knowledge acquired through social interactions with its neighbours. Each particle in a swarm is specified by its position *p*_pso_, its velocity *v*_pso_, and its memory of the best solution achieved by it so far *p*_pso_^*b*^. Another memory element *g* ^*b*^ denotes the best solution attained thus far by the swarm and is shared among all particles.

The position of a particle signifies the vector containing all the parameters of an RNN instance. The fitness of a particle is calculated using either (8) or (7), depending upon whether the decoupled strategy has been implemented or not, respectively. In other words, if someone chooses not to use the decoupled strategy, then the quality of the solution is determined by (7). However, since, in the decoupled strategy, each gene is dealt with separately, the quality of a solution is determined by (8), and we have used this only. For each generation, the updated position *p*_pso_′ and velocity *v*_pso_′ of a particle for the next generation are calculated based on its best solution achieved so far and the best solution obtained by the entire swarm thus far. Hence,

(10) vpsoi′=w⊗vpsoi+r1c1⊗ppsoib−ppsoi+r2c2⊗gb−ppsoi,

(11) ppsoi′=ppsoi+vpsoi′,

where *w* is the inertia weight term, and it controls the dynamic balance between exploration and exploitation undertaken by a particle. Again, *r*_1_ and *r*_2_ are random numbers in the range [0,1] and usually *c*_1_ = *c*_2_ = 2. The terms *r*_1_ *c*_1_ and *r*_2_ *c*_2_ determine the effect (on the particle velocity) of the best solutions achieved by a particle and the swarm, respectively. The terms are all in a matrix format and thus it is sensible to point out that elementwise multiplications are necessary here and have been symbolised by ⊗.

BA has been recently formulated by Yang based on the echolocation property of real bats [59, 60]. In BA, the virtual bats locate food and inform others about the food source with the help of sound waves. The virtual bats are assumed to have the ability to modulate the sound waves according to the need, that is, locating food/prey or communicating with others. The virtual bats are also scattered in the search space, with the position of each virtual bat denoting possible solutions. A virtual bat is completely specified by its position *p*_ba_, its velocity *v*_ba_, loudness *A*, and frequency *f*. A memory element *p*_best_ stores the position of the best food source found so far. The velocity and position of a virtual bat are updated according to the following equations:

(12) fi=fmin+μ⊗fmax−fmin,vbai′=vbai+fi⊗pbest−pbai,pbai′=pbai+vbai′,

where *μ* ∈ [0,1] is a random vector. In this investigation, if standalone BA had been used, then the pertinent values would have been *f*_min_ = 0 and *f*_max_ = 1. At the outset, each virtual bat is arbitrarily allocated a frequency from [*f*_min_, *f*_max_], drawn uniformly. This frequency term controls the movement of the virtual bats in the search space, similar to what the inertia weight term does in case of PSO, as can be seen in (10). There are various ways of updating the inertia weight for PSO.

In this paper, we have proposed a new technique based on the update technique of frequency of virtual bats in BA. We have proposed to update the inertia weight *w* in PSO in each iteration using the following equation:

(13) $$\begin{array}{c} w_{i}=w_{min}+\mu ⊗\left(\;w_{max}-w_{min}\;\right), \end{array}$$

where *μ* ∈ [0,1] is also a random vector. We have assumed *w*_min_ = 0 and *w*_max_ = 1. In each iteration, for each particle, an inertia weight is drawn uniformly from [*w*_min_, *w*_max_]. This somewhat counterbalances the problem of being trapped at local minima, which is one of the few but major shortcomings of PSO. The proposed novel BAPSO algorithm, with the new inertia weight update technique, used for the particular problem domain dealt with herein, has been able to produce better results than individual PSO or BA algorithms (as suggested by other investigations carried out by the authors).

Another change, inspired by the virtual bats in BA that has been incorporated in the proposed BAPSO algorithm, is the initialization of the velocity vector of each particle to zero instead of a random vector. The authors observe that this might help in preventing the particles from having an initial unguided velocity that may divert them away from a potential optimal solution in the search space.

## 4. Experimental Results and Discussion

Owing to the stochastic nature of the proposed framework implemented, it is quite normal that, for any given temporal genetic expression dataset, the predicted GRN would vary in its topology for each independent solution generated. To circumvent this problem, we have employed, in this investigation, a collaborative learning method. We have performed *L* independent experiments and have stored in memory each *L* inferred GRN. Additionally, a selection scheme has been implemented based on a plausibility score ps_*i*,*j*_, assigned to each edge *e*_*i*,*j*_, as given below. This has been done to identify the most consistent predicted edges for the construction of the final GRN:

(14) $$\begin{array}{c} {ps}_{i,j}=\frac{1}{L}\overset{L}{\underset{1}{\sum }}g_{i,j}. \end{array}$$

In (14), *g*_*i*,*j*_ ∈ *G* and ps_*i*,*j*_ ∈ [0,1]. On the evaluation of ps_*i*,*j*_ for all *i* and *j*, the final predicted network, thus, can be generated and represented by *G*_*F*_ = [*g*_*i*,*j*_^*f*^]_*N*×*N*_. Whether the value of a particular element *g*_*i*,*j*_^*f*^ is 0 or 1 can be evaluated using the following relation:

(15) $$\begin{array}{c} g_{i,j}^{f}=\left\{\begin{array}{cc} 1, & if\;\;{ps}_{i,j}\geq \alpha ,\\ 0, & otherwise. \end{array}\right. \end{array}$$

In the above equation, *α* is a threshold defined for the purpose of inclusion of an interaction in a GRN. In other words, it governs whether an edge is included in *G*_*F*_ or omitted altogether. In order to estimate the accuracy of the proposed methodology, we have compared *G*_*F*_ with the original GRN, denoted by *G*_*O*_. In addition, we have quantitatively compared the results of the proposed framework with those from the contemporary literature based on certain metrics. Before explaining the metrics, it would be prudent to mention that an edge can be characterised into four types: true positive (TP), false positive (FP), true negative (TN), and false negative (FN), with their mathematical definitions as follows:

 TP: if *g*_*i*,*j*_^*o*^ = 1 and *g*_*i*,*j*_^*f*^ = 1; TN: if *g*_*i*,*j*_^*o*^ = 0 and *g*_*i*,*j*_^*f*^ = 0.
 FP: if *g*_*i*,*j*_^*o*^ = 0 and *g*_*i*,*j*_^*f*^ = 1; FN: if *g*_*i*,*j*_^*o*^ = 1 and *g*_*i*,*j*_^*f*^ = 0.

Next, we have defined the metrics one by one based on which the proposed methodology can be evaluated.

*(i) True Positive Rate (TPR)/Sensitivity/Recall*. This signifies the fraction of the total number of existing edges in the original network, correctly predicted in the inferred network.

*(ii) True Negative Rate/Specificity (SPC)*. This signifies the fraction of the total number of nonexistent edges in the original network, correctly identified as nonexistent in the inferred network as well.

*(iii) False Positive Rate (FPR)/Complimentary Specificity*. This signifies the fraction of the total number of nonexistent edges, incorrectly predicted in the inferred network.

*(iv) False Negative Rate (FNR)/Complimentary Sensitivity*. This signifies the fraction of the total number of nonexistent edges in the original network, incorrectly guessed in the predicted network.

*(v) Positive Predictive Value (PPV)/Precision*. This signifies the fraction of the total number of inferred edges, which is correct.

*(vi) False Discovery Rate (FDR)/Complimentary Precision*. This signifies the fraction of the total number of inferred edges, which is incorrect.

*(vii) Accuracy (ACC)*. This signifies the fraction of the total number of all possible connections, in the original network, truly predicted.

*(viii) F-Score*. This signifies the harmonic mean of the precision and sensitivity.

 Mathematically speaking,

(16) TPR=TPTP+FN,SPC=TNFP+TN,FPR=FPFP+TN=1−SPC,FNR=FNTP+FN=1−TPR,PPV=TPTP+FP,FDR=FPTP+FP=1−PPV,ACC=TP+TNTP+FP+FN+TN,F=2TP2TP+FP+FN.

The statistical BAPSO methodology has been applied primarily on an artificial network (4 genes). Subsequently, we have applied the proposed methodology on a group of experimental (*in vivo*) time series genetic expression datasets of a real-world network (the 8-gene* E. coli* SOS DNA repair network). Finally, we have experimented with two networks extracted from the genome of* Saccharomyces cerevisiae* (10 genes) and* Escherichia coli* (20 genes) with the help of GeneNetWeaver [63]. Additionally, we have implemented our proposed algorithm on each of these networks, but with half the number of time points initially used for experimentation. This has been done to observe the accuracy of the method if a lesser number of time points are available for training.

All the simulations have been run on a desktop computer running on a 3.4 GHz Intel Core i7 processor with 8 GB 1600 MHz RAM. The codes have been run on Matlab 2014a, running in a Windows 7 64-bit environment.

### 4.1. Artificial Network

**Figure 3:**
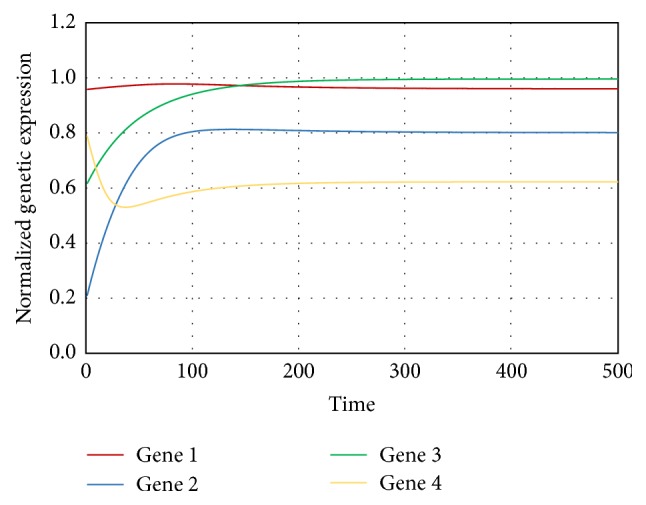
Network dynamics used for training the proposed model. The four lines represent the expression profile of the four genes.

This artificial network consists of 4 genes and 8 interactions. This network has been extensively studied by researchers for the purpose of preliminary validation of their methodologies with respect to reverse engineering of GRNs from time series genetic microarray data [32, 34, 46]. The time series expression data have been generated using (3). The parameters and their related values necessary for calculations have been given in Table 1. The generated expression profiles have been shown in Figure 3. We have assumed Δ*t* = 0.1 for this case and have generated 500 time points with the help of (3).

However, in real-world experiments, such a high number of time points do not typically exist. Therefore, we have sampled the data evenly into 50 time points and have implemented our proposed methodology on the sampled dataset. Further, we have evenly sampled this reduced dataset to produce another dataset with 25 time points.

**Figure 4:**
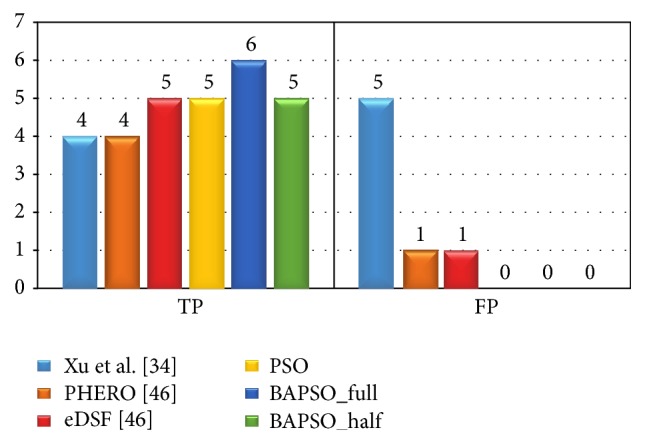
True positive (TP) and false positive (FP) counts obtained by the proposed BAPSO model, compared with those obtained by Xu et al. [34] and Kentzoglanakis and Poole [46] and PSO. The results of the BAPSO model have been presented for two datasets: one with 50 time points, represented as BAPSO_full, and the other with 25 time points, represented as BAPSO_half.

The reverse engineering initiative involves *L* = 10 independent experiments. We have conducted each experiment with a swarm population of ^4^ *C*_*m*_ (where *m* = 1,2, 3,4) particles and 100000 iterations. The statistical properties of the final inferred network have been shown in Figure 4, for *α* = 0.9. Utilising just a single time series, the results show marked improvement over those published by Xu et al. [34] and Kentzoglanakis and Poole [46], with respect to both true and false positives albeit with a stricter threshold than that used by the authors (*σ* = 0.5) in [46]. The average MSE for the experiments are ~10^−6^ and ~10^−5^ for the dataset with 50 time points and the one with 25 time points, respectively. The computational times for both experiments are 15.6 minutes and 8.6 minutes, respectively.

### 4.2. *E. coli* DNA SOS Repair Network

**Figure 5:**
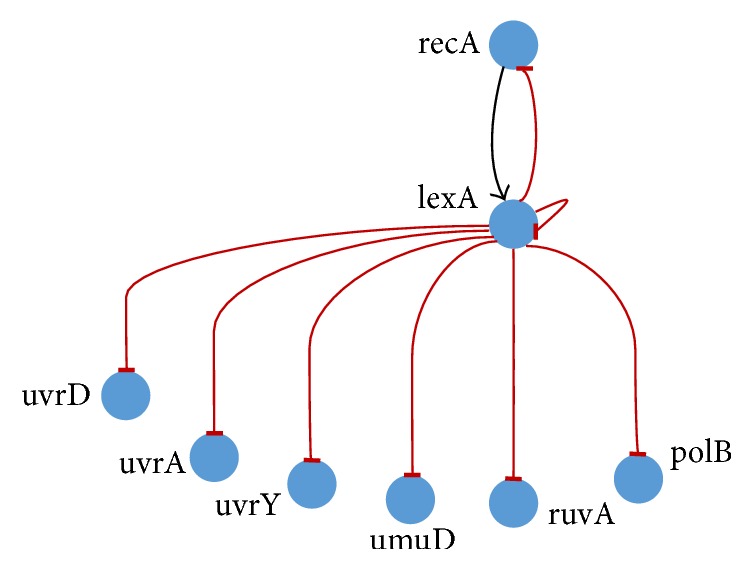
The original structure of the SOS DNA repair transcriptional network of* E*.* coli*.

In this section, the proposed methodology for reverse engineering of GRNs from temporal genetic expression profiles has been employed to identify the causal relationships among the genes from an* in vivo* (experimental) microarray dataset. The said dataset summarises the dynamics of the well-illustrated transcriptional network involved in the SOS DNA repair mechanism of* E. coli* studied experimentally by Ronen et al. [64]. The study included eight genes heavily involved in the SOS repair mechanism: recA, lexA (the master repressor), uvrA, uvrD, uvrY, umuD, ruvA, and polB. The original network has been shown in Figure 5. Four experimental datasets had been generated using two different UV light intensities on* E. coli* (for experiments 1 and 2: UV intensity used → 20 Jm^−2^; for experiments 3 and 4: UV intensity used → 5 Jm^−2^). In each of the experiments, expression data had been observed for 50 time points each using temporal resolution of 6 minutes. These datasets are one of the most useful ones concerning the qualitative investigations on computational methods for reconstruction of GRNs from time series genetic expression data (which for ready reference is at http://wws.weizmann.ac.il/mcb/UriAlon/sites/mcb.UriAlon/files/uploads/DownloadableData/sosdata.zip).

In this case also, *L* = 10 independent experiments have been performed for each of the four datasets. A swarm population of _ _^8^ *C*_*m*_^ ^ (where *m* = 1,2, 3,4) has been used with a maximum number of iterations set to 5000. The expression value of each gene in each dataset at the first time point is zero and hence has been ignored. Subsequently, all expression values have been normalised to the range [0,1]. The dataset thus contains 49 time points. We have also taken alternative time points and created a truncated dataset with 25 points. The statistical properties of the predicted GRN in each experiment with a plausibility score threshold, set at *α* = 0.9, have been shown in Table 2.

Table 3 displays a quantitative comparison of the characteristics of the predicted GRNs (with a plausibility score threshold, *α* = 0.9) with those presented in a recent investigative work (with an inclusion threshold, *σ* = 0.9) [46]. The proposed methodology is consistent regarding the number of true (and false) positives predicted compared to results presented in [46] for different experimental datasets. The method proposed in [46] fails to identify any true positive in the fourth experiment whereas the framework proposed in this paper does not fail to identify true positives for any experiment. However, we have to concede that the proposed framework cannot match the isolated best result obtained by the eDSF model [46] in the case of the second experiment. However, it may be noted that the regulatory relationship between recA and lexA was not inferred in any of the experiments conducted by Kentzoglanakis and Poole [46], whereas the proposed methodology can identify this particular interaction in one of the four experiments. This suggests that it probably is among a few proposed computational frameworks that are capable of identifying all the regulatory interactions present in the SOS response network of* E. coli*. A qualitative comparison of several such methodologies [19, 34, 46, 61, 62] is given in Table 4. The performance of the methodology with half the number of time points is also admirable and has been included in Tables 2 and 3.

### 4.3. 10-Gene Network Extracted from GeneNetWeaver (GNW)

**Figure 6:**
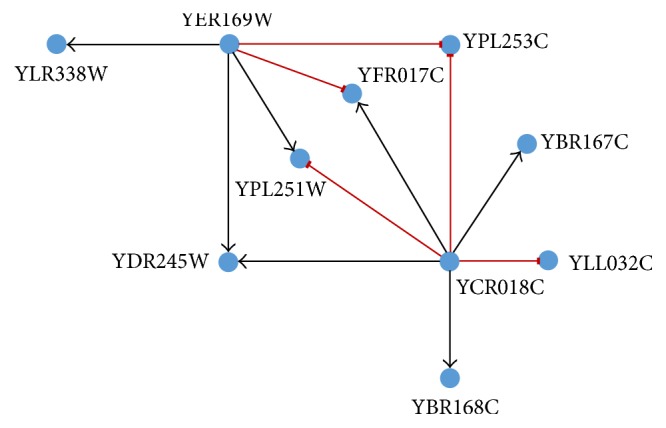
Network architecture extracted from GNW to validate the proposed framework as used in [46].

The* in silico* datasets have been extracted from the genome of yeast and* E. coli* stored in GNW [63]. First, we have considered the yeast network, made up of 10 genes and 12 genetic interactions as shown Figure 6. We have generated the network dynamics with the help of GeneNetWeaver [63] in keeping with DREAM4 settings [65]. Two sets of genetic expression data have been generated, one with 50 time points and the other with 25 time points (taking the alternate time points of the former). The number of independently generated solutions is *L* = 10. Since there are 10 genes in the GRN, the problem has been divided into 10 subproblems, each with 12 parameters to optimise. For each of the suboptimization problems, a swarm population of _ _^10^ *C*_*m*_^ ^ (where *m* = 1,2, 3,4) has been used, and the maximum number of iterations has been set to 10000.

**Figure 7:**
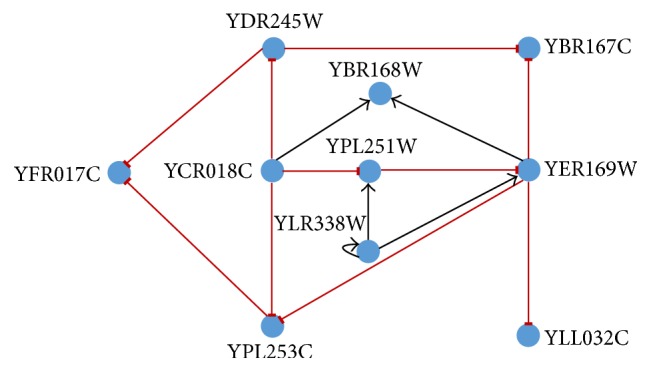
Inferred network obtained by the proposed model for 50 time points.

**Figure 8:**
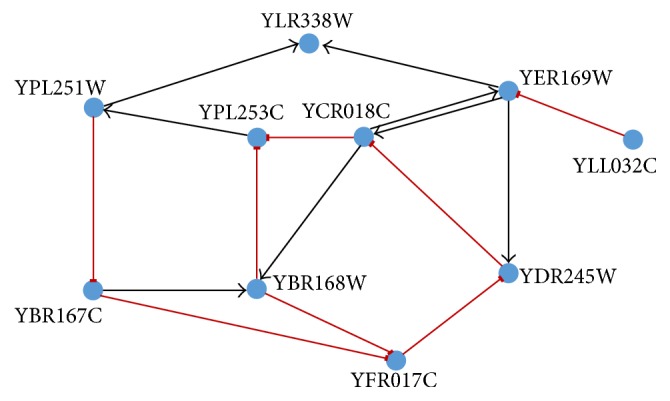
Inferred network obtained by the proposed model for 25 time points.

The results achieved in this experiment have been summarised in Table 5. The proposed methodology can correctly predict 5 (for *α* ≥ 0.9) out of a possible 12 interactions present in the original network using the dataset with 50 time points. The proposed methodology can also correctly predict 4 (for *α* ≥ 0.9) out of a possible 12 interactions present in the original network using the dataset with 21 time points. With the increase in *α*, the number of incorrect predictions goes down from 13 to 9, increasing the accuracy from 81% to 84%, and from 15 to 11, increasing the accuracy from 77% to 81%, respectively, in the two cases. The final predicted GRNs for the two cases have been shown in Figures 7 and 8.

The results have been compared with previous similar work published in [46] and have been shown in Table 6. The proposed methodology indicates improvement from the perspective of true predictions. Even for the most stringent value of the threshold, that is, *α* = 1, the number of true predictions is significantly more (5 compared to 3) with 50 time points and still better (4 compared to 3) with 25 time points. The true positive rate and the precision of the predicted network are almost always better than the compared network. Considering the nature of the inferred relationships (whether activation or repression), the proposed methodology has correctly identified the nature of 80% of the predicted relationships.

### 4.4. 20-Gene Network Extracted from GeneNetWeaver (GNW)

**Figure 9:**
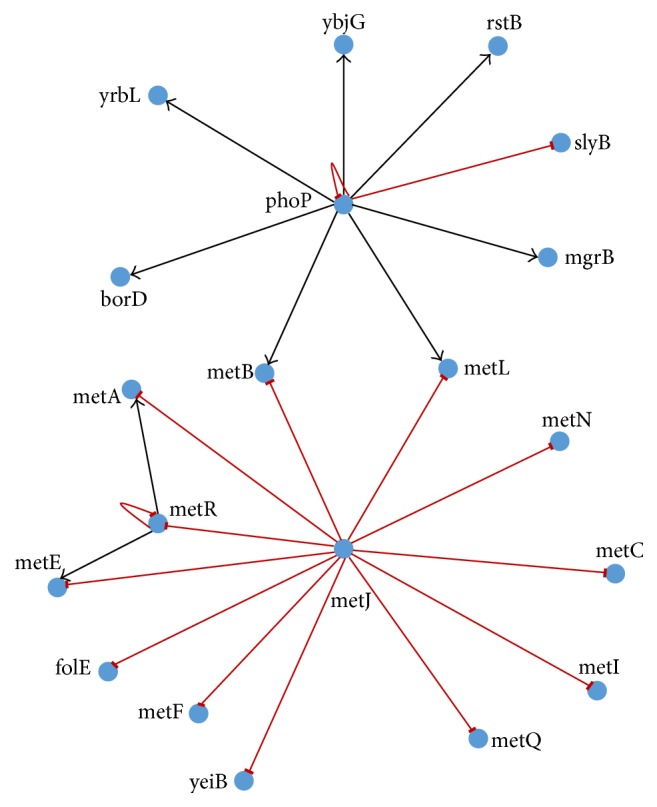
Original 20-gene network extracted from the genome of* E. coli* stored in GNW.

The second network extracted from GNW is a 20-gene network consisting of 24 interactions. The datasets for this network have been generated using the same settings as the previous one. We have generated *L* = 10 independent solutions. There are 20 genes in this GRN, and hence the problem has been divided into 20 subproblems, each with 22 parameters to optimise. For each of the suboptimization problems, a swarm population of _ _^20^ *C*_*m*_^ ^ (where *m* = 1,2, 3,4) has been used, and the maximum number of iterations has been set to 10000. The original network is shown in Figure 9.

The proposed RNN based framework does not scale up with the size of the GRN, efficiently. For the 20-gene network considered here, it was able to predict only 3 out of a possible 24 interactions correctly, but with a large number of false positives. Fascinatingly, however, the proposed method can correctly predict 5 interactions out of a possible 24, with 2 less false positives. The predicted correct relations have been shown in Table 7.

## 5. Conclusion

In this paper, we have investigated the domain of reconstruction of GRNs from time series microarray datasets with modifications in the existing methodologies. For this purpose, we have implemented a decoupled technique based on the novel BAPSO algorithm, the fundamental mathematical theory of combination, and RNN. The main objective of the investigation is to detect the biologically relevant GRNs from the large discrete network architecture search space. Prior knowledge and the fundamental theory of combination have been used for the purpose of reducing the dimension of the optimisation problem, thus reducing the computational load. Also, the proposed methodology ensures a higher probability of identifying a more biologically relevant network as it searches all possible candidate architectures (i.e., all possible combinations).

The proposed novel hybrid swarm intelligence scheme, BAPSO, has been implemented in the present investigation to train the RNN model parameters and the results obtained show that the predicted networks reproduce the dynamics of the given dataset to a better extent for small-scale GRNs. The results suggest that the proposed decoupled reverse engineering approach is robust and consistent with respect to the number of correct and incorrect predictions while using different types of microarray datasets (synthetic,* in silico*, and* in vivo*) for most of the small-scale GRNs studied in the contemporary literature.

However, it is an entirely different scenario for medium-scale networks (20 genes). The methodology fails to reproduce any of the successes it had against smaller GRNs. There are too few true predictions and a large number of incorrect predictions. The methodology, implemented in this paper, thus, needs to be enriched further by studying its performance in larger networks. This provides a vital scope for further research.

Also, the assumption of the value of the threshold, *α*, is based on the knowledge of the final network to be obtained. In real-world cases, where the final GRN is not known, the setting of a suitable threshold for the ensemble learning scheme used in this work needs further research. Additionally, the reduction in false positives is also an important research endeavour for the future.

Another point to be noted in this context is the performance of the methodology with a lesser number of time points, half to be exact. The results indicate that a 50% reduction in the number of time points leads to only a small drop in accuracy of the predicted models, a maximum of just over 15% in the worst-case scenario. However, interestingly, the methodology slightly improves upon the poor results obtained for the 20-gene GRN, with a lesser number of time points available.

This also provides an opportunity for future research into the prediction of GRNs from genetic expression profiles with lesser time points and will surely help to reduce time and cost of data generation in the future.

## Figures and Tables

**Table 1:** RNN model parameters [32, 34].

*w*_*i*,*j*_				*b*_*i*_	*c*_*i*_
20	−20	0	0	0	10
15	−10	0	0	−5	5
0	−8	12	0	0	5
0	0	8	−12	0	5

**Table 2:** Results of the *E. coli* experiments with all the available time points.

Dataset	TP	TN	FP	FN	TPR	SPC	FPR	FNR	PPV	FDR	ACC	*F*1-score	Graph edges	MSE	CPU time
BAPSO_full															
1	7	46	9	2	0.78	0.84	0.16	0.22	0.44	0.56	0.83	0.56	16	0.0157	16.9 m
2	7	40	15	2	0.78	0.73	0.27	0.22	0.32	0.68	0.73	0.45	22	0.0114	16.9 m
3	7	45	10	2	0.78	0.82	0.18	0.22	0.41	0.59	0.81	0.54	17	0.0147	16.9 m
4	4	43	12	5	0.44	0.78	0.22	0.56	0.25	0.75	0.73	0.32	16	0.0147	16.9 m

BAPSO_half															
1	7	38	17	2	0.78	0.69	0.31	0.22	0.29	0.71	0.70	0.42	24	0.0042	9.1 m
2	8	40	15	1	0.89	0.73	0.27	0.11	0.35	0.65	0.75	0.50	23	0.0025	9.2 m
3	6	43	12	3	0.67	0.78	0.22	0.33	0.33	0.67	0.77	0.44	18	0.0048	9.1 m
4	4	40	15	5	0.44	0.73	0.27	0.56	0.21	0.79	0.69	0.29	19	0.0039	9.1 m

**Table 3:** Comparison of results obtained from the *E. coli* experiments with [46].

Dataset	TP				FP			
	eDSF [46]	PSO	BAPSO_full	BAPSO_half	eDSF [46]	PSO	BAPSO_full	BAPSO_half
1	3	5	**7**	**7**	10	9	**9**	**17**
2	8	4	**7**	**8**	5	10	**15**	**15**
3	4	5	**7**	**6**	9	8	**10**	**12**
4	0	3	**4**	**4**	9	8	**12**	**15**

**Table 4:** Comparison with contemporary research [46] for the *E. coli* experiments.

Known interactions	Predictions by						
	[13]	[61]	[34]	[46]	[62]	PSO	BAPSO
lexA → lexA	Yes	Yes	No	Yes	Yes	Yes	Yes
lexA → recA	Yes	Yes	Yes	Yes	Yes	Yes	Yes
recA → lexA	Yes	Yes	No	No	No	Yes	Yes
lexA → uvrA	Yes	Yes	Yes	Yes	Yes	Yes	Yes
lexA → uvrD	No	No	Yes	Yes	Yes	Yes	Yes
lexA → uvrY	No	No	No	Yes	No	Yes	Yes
lexA → umuD	No	Yes	Yes	Yes	Yes	Yes	Yes
lexA → ruvA	No	No	No	Yes	No	Yes	Yes
lexA → polB	No	No	Yes	Yes	Yes	Yes	Yes

Spurious edges (FP)	5	10	2	5	3	10	9

Precision (PPV)	0.44	0.33	0.71	0.62	0.70	0.47	0.44

**Table 5:** Results for the yeast dataset extracted from GNW with 50 time points and 25 time points represented as BAPSO_full and BAPSO_half.

*α*	TP	TN	FP	FN	TPR	SPC	FPR	FNR	PPV	FDR	ACC	*F*1-score	Graph edges	MSE	CPU time
BAPSO_full															
0.5	6	75	13	6	0.50	0.85	0.15	0.50	0.32	0.68	0.81	0.39	19	0.0034	27.4 minutes
0.6	6	75	13	6	0.50	0.85	0.15	0.50	0.32	0.68	0.81	0.39	19		
0.7	6	76	12	6	0.50	0.86	0.14	0.50	0.33	0.67	0.82	0.40	18		
0.8	6	76	12	6	0.50	0.86	0.14	0.50	0.33	0.67	0.82	0.40	18		
0.9	5	78	10	7	0.42	0.89	0.11	0.58	0.33	0.67	0.83	0.37	15		
1.0	5	79	9	7	0.42	0.90	0.10	0.58	0.36	0.64	0.84	0.38	14		

BAPSO_half															
0.5	4	73	15	8	0.33	0.83	0.17	0.67	0.21	0.79	0.77	0.26	19	0.0048	14.7 minutes
0.6	4	73	15	8	0.33	0.83	0.17	0.67	0.21	0.79	0.77	0.26	19		
0.7	4	74	14	8	0.33	0.84	0.16	0.67	0.22	0.78	0.78	0.27	18		
0.8	4	74	14	8	0.33	0.84	0.16	0.67	0.22	0.78	0.78	0.27	18		
0.9	4	76	12	8	0.33	0.86	0.14	0.67	0.25	0.75	0.80	0.29	16		
1.0	4	77	11	8	0.33	0.88	0.13	0.67	0.27	0.73	0.81	0.30	15		

**Table 6:** Comparison of BAPSO results for the GNW dataset of yeast with PSO and eDSF [46].

Threshold	PSO	BAPSO_full	BAPSO_half	eDSF [46]	PSO	BAPSO_full	BAPSO_half	eDSF [46]
	TPR				FPR			
0.5	0.42	**0.50**	0.33	0.50	0.14	**0.15**	0.17	0.19
0.6	0.42	**0.50**	0.33	0.42	0.13	**0.15**	0.17	0.14
0.7	0.42	**0.50**	0.33	0.42	0.08	**0.14**	0.16	0.14
0.8	0.42	**0.50**	0.33	0.42	0.08	**0.14**	0.16	0.15
0.9	0.42	**0.42**	0.33	0.42	0.06	**0.11**	0.14	0.13
1.0	0.42	**0.42**	0.33	0.25	0.05	**0.10**	0.13	0.08

	Accuracy				Graph edges			
0.5	0.81	**0.81**	0.77	Not available	17	**17**	19	19
0.6	0.82	**0.81**	0.77		16	**16**	19	19
0.7	0.86	**0.82**	0.78		12	**12**	18	18
0.8	0.86	**0.82**	0.78		12	**12**	18	18
0.9	0.88	**0.83**	0.80		10	**10**	16	15
1.0	0.89	**0.84**	0.81		9	**9**	15	14

**Table 7:** True positives obtained for the GRN consisting of 20 genes.

Technique	BAPSO_full	BAPSO_half
Correct interactions	metJ → metN, metJ → folE, and metR → metL	metJ → metN, metJ → metC, metJ → metQ, phoP → yrbL, and phoP → borD
Computational time	3.2 hrs	1.7 hrs
MSE	0.0031	0.0034
